# Association between systemic inflammation and overactive bladder: The potential mediating role of obesity-related anthropometric indices

**DOI:** 10.1097/MD.0000000000050574

**Published:** 2026-09-11

**Authors:** Hao Du, Lei Wang, Honglan Zhou

**Affiliations:** a Department of Urology II, The First Hospital of Jilin University, Changchun, China.

**Keywords:** cross-sectional study, intermediary analysis, NHANES, overactive bladder, the aggregate index of systemic inflammation

## Abstract

Systemic inflammation is increasingly implicated in the development of chronic diseases, yet the specific association between the Aggregate Index of Systemic Inflammation (AISI) – a novel composite inflammatory marker – and overactive bladder (OAB) remains poorly understood. This study aimed to elucidate the association between AISI levels and OAB prevalence and to explore the statistical mediating effect of anthropometric indicators within the potential inflammation-metabolism pathway. This cross-sectional study utilized data from the National Health and Nutrition Examination Survey (NHANES) 2009–2018, including 18,601 participants aged ≥ 20 years. Weighted multivariate logistic regression and smooth curve fitting were employed to evaluate the association between the logarithmic AISI (LN-AISI) and OAB. Subgroup analyses and interaction tests were conducted to assess robustness. Furthermore, an exploratory statistical mediation analysis was performed to quantify the extent to which body mass index (BMI), relative fat mass (RFM), weight-adjusted-waist index (WWI), and body roundness index (BRI) statistically mediate this association. Participants in the highest quartile of LN-AISI exhibited 21% higher odds of OAB compared to the lowest quartile (OR 1.21, 95% CI: 1.02–1.44, *P* = .0349) after adjusting for covariates. Smooth curve fitting revealed a nonlinear dose–response relationship; notably, above a data-driven threshold of LN-AISI > 5.71, each unit increase was associated with 50% higher odds of OAB (OR 1.50, 95% CI: 1.32–1.70, *P* < .0001). This association was consistent across most subgroups but modified by age. Mediation analysis suggested that anthropometric indicators statistically mediated the AISI-OAB association, with mediation proportions of 31.3% for BMI, 36.2% for RFM, 26.4% for WWI, and 37.1% for BRI. Elevated AISI is associated with higher odds of OAB in a nonlinear, dose–dependent manner, particularly among older adults. Obesity-related anthropometric indices appear to statistically mediate this association in our cross-sectional sample. These findings suggest that managing systemic inflammation and body weight may represent associated factors worth exploring in future preventive research on OAB. However, given the cross-sectional design, these results represent statistical associations rather than confirmed causal mechanisms, warranting further longitudinal validation.

## 1. Introduction

Overactive Bladder (OAB), a typical lower urinary tract dysfunction, has a core clinical symptom of sudden onset of urinary urgency, which is often accompanied by concomitant symptoms such as urinary frequency and increased nocturia. Its diagnosis requires the strict exclusion of urinary tract infections and other organic pathologies.^[1]^ US adult population-based studies demonstrate comparable OAB prevalence between genders: 7.7% in males versus 6.7% in females.^[2]^ European cohort studies revealed significant gender disparity: 10.8% prevalence in males versus 12.8% in females.^[3]^ This gender difference tended to widen significantly in older age groups.^[4]^ It is important to note that OAB is not only an “invisible killer” of patients’ quality of life, but also a multidimensional public health challenge due to the long-term healthcare expenditures and productivity losses caused by the disease.^[5]^ Identifying modifiable risk factors associated with OAB is of great public health value for the development of precise intervention programs and the optimization of health resource allocation. However, consensus remains elusive regarding primary contributing factors, and studies have revealed that its occurrence is associated with multisystem dysfunction. Specifically, neuromodulatory abnormalities,^[6]^ chronic inflammatory responses,^[7]^ imbalances in body fat metabolism,^[8]^ and psychosomatic disorders^[9]^ have been implicated in the epidemiology of the condition.

Recent studies have highlighted the association between chronic inflammation and urinary system homeostasis.^[7,10-12]^ As an innovative composite index for systemic inflammation, the aggregate index of systemic inflammation (AISI) integrates peripheral counts of neutrophils, monocytes, lymphocytes, and platelets to quantitatively reflect the body’s inflammatory burden.^[13]^ AISI’s clinical utility has been established across multiple pathologies, including hypertension, chronic kidney disease, and idiopathic pulmonary fibrosis.^[14-19]^ However, the relationship between AISI and OAB remains underexplored. Therefore, elucidating its epidemiological connection will not only expand our understanding of the role of chronic inflammation in OAB, but may also lay a theoretical foundation for future biomarker research.

In addition, obesity has been established as a significant correlate of OAB.^[20]^ Anthropometric measures including body mass index (BMI), relative fat mass (RFM), weight-adjusted waist index (WWI), and body roundness index (BRI) serve as independent predictors of OAB.^[21,22]^ Concurrently, chronic inflammatory states are closely correlated with adiposity and lipid metabolism dysregulation.^[23,24]^ These findings position obesity as a potential statistical mediator linking inflammation and OAB. Based on the above epidemiological evidence, the present study aimed to systematically evaluate the potential association between AISI and OAB by integrating data from 5 cycles of the NHANES database from 2009-2018. We hypothesized that elevated AISI levels are associated with a higher prevalence of OAB, and that this relationship is statistically mediated by anthropometric indices (BMI, RFM, WWI, and BRI). By employing an exploratory mediation analysis, this study seeks to provide epidemiological insights into the complex interplay between systemic inflammation, obesity, and OAB, while explicitly acknowledging that causal pathways cannot be confirmed within this cross-sectional framework.

## 2. Materials and methods

### 2.1. Data source and study population

This study utilized the National Health and Nutrition Examination Survey (NHANES) database, established by the US Centers for Disease Control and Prevention (CDC). The database provides multifaceted data, including anthropometric measurements, laboratory analyses, and questionnaire responses.

**Figure 1.**
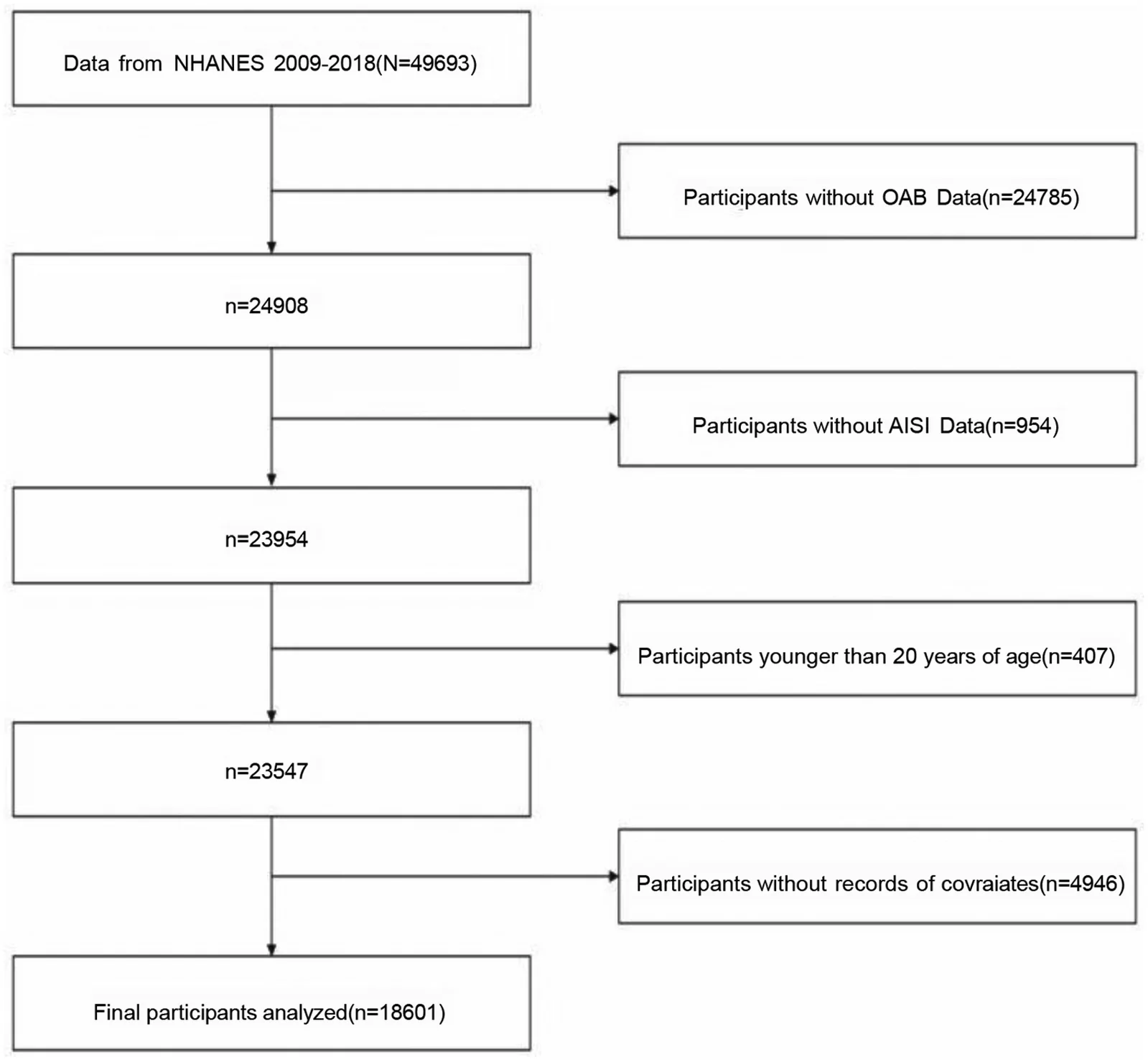
Flowchart of participant selection. AISI = Aggregate Index of Systemic Inflammation, NHANES = National Health and Nutrition Examination Survey, OAB = overactive bladder.

The study protocol adhered to the ethical guidelines of the Declaration of Helsinki, with written informed consent obtained from all participants following approval by the Institutional Review Board (IRB). Data were extracted from 5 NHANES cycles (2009–2018), with an initial cohort of 49,693 eligible participants identified through screening. Subsequently, an effective sample size of 18,601 was finalized by stepwise exclusion of the following based on preset exclusion criteria: missing OAB diagnostic information (24,785 cases); incomplete AISI data (954 cases); under age 20 years (407 cases); and missing key covariates (4946 cases). Therefore, we directly excluded samples containing missing values to handle the missing data. A detailed flowchart outlining the participant screening process and exclusion criteria is provided in Figure 1.

### 2.2. AISI measurement

AISI is derived from complete blood count (CBC) parameters to assess systemic inflammatory status. Laboratory testing for this indicator is performed using Beckman Coulter Molecular Weight Quantification (MWQ) technology, which integrates an automated sample dilution system, an intelligent mixing device, and a single-beam photometer (dedicated to hemoglobin testing) for accurate quantitative analysis. All hematological analyses at NHANES Mobile Examination Centers utilized the Beckman Coulter DxH 800 system, which delivers precise differential counts of leukocyte subpopulations. The resulting AISI formula is (Neutrophils (NEU) * Platelets (PLT) * Monocytes (MONO))/ Lymphocytes (LYM).

### 2.3. Assessment of OAB

The diagnostic criteria for OAB mainly focus on urge incontinence and nocturia as the core clinical diagnosis. In this study, a standardized symptom assessment protocol was constructed by integrating the NHANES Urologic Disease Specialty Questionnaire module to quantify symptom severity using 3 standardized items: Have you experienced urgent urinary leakage preventing timely toilet access in the past year? What was the frequency of these episodes? How many nightly awakenings for urination occurred in the past month? Symptom quantification was standardized using the clinically validated Overactive Bladder Syndrome Symptom Scale (OABSS).^[25]^ The diagnosis of OAB is confirmed when the sum of the 2 sub-scores of urge incontinence and nocturia reaches the diagnostic threshold (≥3 points) according to the most recent International Continence Society (ICS) protocols. It is important to note that OAB classification in this study was entirely based on self-reported questionnaire data, without objective urodynamic confirmation or adjustments for specific urological medications. Complete scoring methodology is detailed in Table S1, Supplemental Digital Content 1.

### 2.4. Covariates

In this study, a comprehensive set of covariates were selected a priori based on established clinical relevance and prior literature to control for the effect of potential confounders on the relationship between AISI and OAB. Continuous variables included age and PIR, while categorical variables encompassed demographic characteristics and health-related variables. Demographic characteristics included gender (male, female), race (Mexican American, other Hispanic, non-Hispanic White, non-Hispanic Black, other race), education level (less than high school, high school, more than high school), and marital status (never married, widowed/divorced/separated, married/living with partner). Health-related variables included smoking (never, ever, current), alcohol use (never, ever, current), hypertension (no/yes), diabetes mellitus (no/yes), and hyperlipidemia (no/yes). Variables are defined in detail in Table S2, Supplemental Digital Content 2.

### 2.5. Statistical analysis

The analysis implemented the NHANES multistage stratified probability sampling design with appropriate sample weighting to maintain national representativeness per CDC analytic guidelines. Because this study pooled 5 consecutive NHANES cycles (2009–2018), combined sample weights were constructed in accordance with the NHANES analytic guidelines by dividing the original 2-year Mobile Examination Center weights (WTMEC2YR) by the number of pooled cycles (i.e., divided by 5) to generate 10-year combined weights. All analyses explicitly incorporated the complex survey design by specifying the masked variance pseudo-stratum (SDMVSTRA) as strata, the masked variance pseudo-primary sampling units (SDMVPSU) as primary sampling units, and the constructed pooled weights as sampling weights, implemented using the survey package in R. For descriptive statistical analysis, participants were stratified by OAB status (present/absent) for baseline characteristic comparisons. Continuous variables were expressed as mean ± standard deviation (SD) and compared using the weighted *t* test, while categorical variables were expressed as frequency (percentage) and analyzed using the chi-square test. Given that AISI exhibits significant skewed distribution characteristics, the study used natural logarithmic transformation (ln-AISI) followed by stratification based on interquartile spacing. In order to systematically analyze the association between ln-AISI and OAB, we constructed 3 weighted logistic regression models, of which model 1 was unadjusted for covariates, model 2 was adjusted for some demographic covariates, including gender, age, and race, and model 3 was a fully-adjusted model, adjusted for gender, age, race, education, marital status, PIR, drinking status, smoking status, diabetes, hypertension, and hyperlipidemia. Subsequently, we used smoothed curve fitting to test for nonlinear trends based on Model III and tested for heterogeneity in the 8 potential effect modifiers (including age, gender, race, smoking, alcohol use, hypertension, hyperlipidemia, and diabetes) rated by subgroup analyses with interactions. Furthermore, an exploratory statistical mediation analysis was conducted to calculate the proportions of mediated effects for body measures such as BMI, RFM, WWI, and BRI using R’s “mediation” package (version 4.3.3; Dustin Tingley). Crucially, mediation analysis within this cross-sectional framework relies on the assumption of temporality and the absence of unmeasured confounding, neither of which can be definitively confirmed here. Therefore, these mediation results must be interpreted strictly as exploratory statistical associations rather than causal pathways. Analyses used R Studio (version 4.3.1; Posit) and EmpowerStats software (version 4.2; X&Y Solutions, Inc.). Two-tailed *P* < .05 defined statistical significance.

## 3. Results

### 3.1. Baseline characteristics of study participants

Table 1 describes the baseline characteristics of the participants, categorizing the 18,601 study participants into a non-OAB group (n = 14,882) versus an OAB group (n = 3719) according to whether they had OAB or not, showing significant differences in demographic and clinical indicators between the 2 groups. Data analysis showed that compared to the non-OAB group, the OAB group exhibited a significant female preponderance (64.61% vs 49.16%), higher age (57.46 vs 45.75 years), and ln-AISI biomarker levels (5.58 vs 5.49), but with a significantly lower household income to poverty ratio (PIR; 2.61 vs 3.16). Further analysis of the characteristics within the OAB group showed that OAB patients were more likely to be female (64.61%), non-Hispanic White (64.22%), married or living with a partner (58.87%), have a high school education or higher (53.39%), have never smoked (52.25%), are currently consuming alcohol (72.70%), have hyperlipidemia (54.96%), and hypertension (59.42%), while not suffering from diabetes (74.05%).

**Table 1:** Baseline characteristics of the study population: NHANES 2009–2018.

Characteristics	Non-OAB (n = 14,882)	OAB (n = 3719)	*P*-value
Age, yr, mean (95% CI)	45.75 (45.21, 46.29)	57.46 (56.65, 58.28)	<.0001
PIR, mean (95% CI)	3.16 (3.09, 3.24)	2.61 (2.49, 2.72)	<.0001
LN-AISI, mean (95% CI)	5.49 (5.47, 5.51)	5.58 (5.54, 5.61)	<.0001
Gender (%)			<.0001
Male	7583 (50.84%)	1419 (35.39%)	
Female	7299 (49.16%)	2300 (64.61%)	
Race (%)			<.0001
Mexican American	2100 (8.18%)	511 (7.53%)	
Other Hispanic	1454 (5.55%)	395 (5.79%)	
Non-Hispanic White	6374 (68.94%)	1437 (64.22%)	
Non-Hispanic Black	2728 (8.94%)	1070 (16.90%)	
Other race	2226 (8.39%)	306 (5.56%)	
Education level (%)			<.0001
Less than high school	2675 (11.42%)	1115 (21.12%)	
High school	3202 (21.39%)	905 (25.48%)	
More than high school	9005 (67.20%)	1699 (53.39%)	
Marital status (%)			<.0001
Never married	2864 (18.32%)	505 (12.05%)	
Widowed/divorced/separated	2710 (16.12%)	1276 (29.08%)	
Married/living with partner	9308 (65.57%)	1938 (58.87%)	
Smoking status (%)			.0006
Never	8732 (58.07%)	1930 (52.25%)	
Former	3260 (23.70%)	1028 (27.75%)	
Now	2890 (18.23%)	761 (20.01%)	
Drinking status (%)			<.0001
Never	1980 (9.75%)	647 (14.13%)	
Former	1292 (7.28%)	576 (13.17%)	
Now	11,610 (82.96%)	2496 (72.70%)	
Hypertension (%)			<.0001
No	9362 (65.93%)	1340 (40.58%)	
Yes	5520 (34.07%)	2379 (59.42%)	
Diabetes (%)			<.0001
No	12,771 (89.07%)	2568 (74.05%)	
Yes	2111 (10.93%)	1151 (25.95%)	
Hyperlipidemia (%)			<.0001
No	9201 (62.03%)	1705 (45.04%)	
Yes	5681 (37.97%)	2014 (54.96%)	

CI = confidence interval, LN-AISI = logarithmic Aggregate Index of Systemic Inflammation, NHANES = National Health and Nutrition Examination Survey, OAB = overactive bladder, PIR = ratio of family income to poverty.

### 3.2. Associations between LN-AISI and risk of OAB

Table 2 systematically presents the association between LN-AISI and OAB. When LN-AISI was analyzed as a continuous variable, all 3 regression models presented statistically significant associations (*P* < .05). Specifically, the odds of OAB increased by 17% for each unit increase in LN-AISI in the fully adjusted model (OR: 1.17, 95% CI: 1.07–1.29, *P* = .0010). To verify the robustness of the association, the study further stratified the LN-AISI into quartiles, and all groups of analyses maintained a significant positive correlation profile: in model 1, each 1-unit increase in the highest quartile of LN-AISI (Q4) was associated with a 31% increase in the odds of OAB, compared with the quartile with the lowest LN-AISI (Q1; OR: 1.31, 95% CI: 1.13–1.53, *P* = .0009). Model 2 adjusted for demographic covariates, resulted in 42% higher odds (OR: 1.42, 95% CI: 1.20–1.69, *P* = .0009). The final fully adjusted model still maintained 40% higher odds (OR: 1.40, 95% CI: 1.10–1.70, *P* = .0032).

**Table 2:** Association between LN-AISI and OAB, NHANES 2009–2018.

	Model 1 Unadjusted OR (95% CI), *P* value	Model 2 Adjusted OR (95% CI), *P* value	Model 3 Adjusted OR (95% CI), *P* value
LN-AISI	1.23 (1.13, 1.34) <.0001	1.28 (1.17, 1.41) <.0001	1.17 (1.07, 1.29) .0010
LN-AISI quartile			
Quartile 1	Ref	Ref	Ref
Quartile 2	0.94 (0.79, 1.11) .4577	1.06 (0.89, 1.27) .5065	1.03 (0.86, 1.23) .7564
Quartile 3	1.01 (0.87, 1.17) .8928	1.11 (0.94, 1.32) .2099	1.03 (0.87, 1.22) .7120
Quartile 4	1.31 (1.13, 1.53) .0009	1.42 (1.20, 1.69) .0002	1.21 (1.02, 1.44) .0349
*P* for trend	.0003	.0001	.0374

Model 1 was unadjusted for variables.

Model 2 was adjusted for gender, age, and race.

Model 3 was adjusted for gender, age, race, education level, PIR, marital status, smoking status, alcohol status, hypertension, diabetes, and hyperlipidemia.

CI = confidence interval, LN-AISI = logarithmic Aggregate Index of Systemic Inflammation, NHANES = National Health and Nutrition Examination Survey, OAB = overactive bladder, OR = odds ratio, PIR = ratio of family income to poverty.

### 3.3. The results of nonlinear relationship

**Figure 2.**
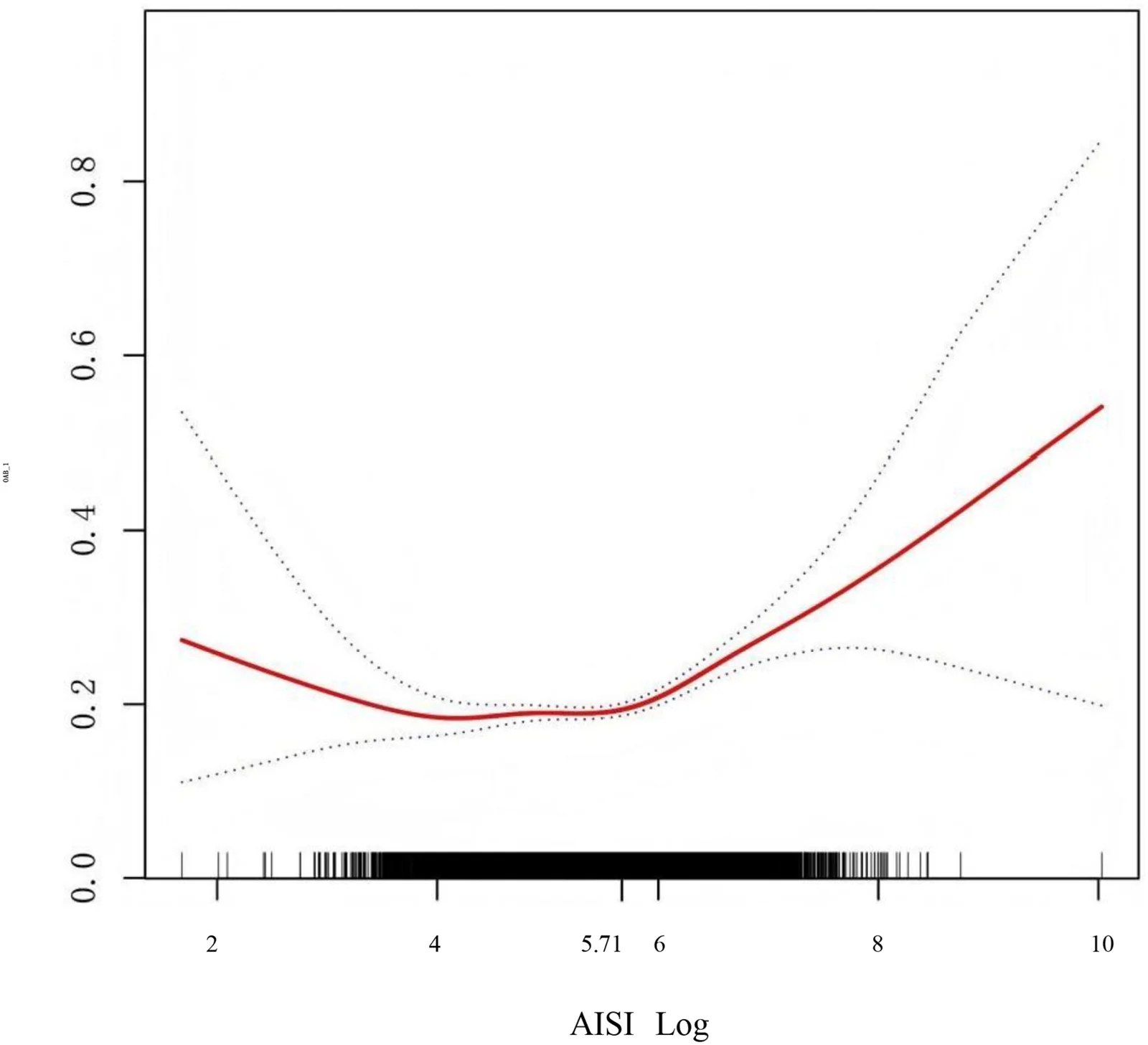
Smoothed curve fitting showing a threshold association between LN-AISI and OAB risk. LN-AISI = logarithmic Aggregate Index of Systemic Inflammation, OAB = overactive bladder.

Smoothed curve fitting revealed a significant threshold effect of LN-AISI on OAB odds. As shown in Figure 2, the curves showed a significant divergence before and after the inflection point after adjusting for all covariates. Threshold analysis identified a data-driven inflection point at LN-AISI = 5.71 (Table 3). When LN-AISI < 5.71, its fluctuation was not statistically associated with OAB odds (OR: 1.00, 95% CI: 0.91–1.10, *P* = .9928). In contrast, above this threshold (LN-AISI ≥ 5.71), each unit increment was associated with a 50% increase in OAB odds (OR: 1.50, 95% CI: 1.32–1.70, *P* < .0001). This nonlinear relationship indicates a potential threshold effect in this population, though the biological or clinical significance of this specific data-driven value requires further investigation.

**Table 3:** Threshold effect analysis of LN-AISI on OAB.

	Adjusted OR (95% CI)	*P* value
Fitting by standard linear model	1.17 (1.11, 1.24)	<.0001
Fitting by 2-piecewise linear model		
Inflection point (5.71)		
LN-AISI < inflection point	1.00 (0.91, 1.10)	.9928
LN-AISI > inflection point	1.50 (1.32, 1.70)	<.0001
*P* for log-likelihood ratio		<.001

CI = confidence interval, LN-AISI = logarithmic Aggregate Index of Systemic Inflammation, OAB = overactive bladder, PIR = ratio of family income to poverty.

### 3.4. Analysis of subgroup

**Figure 3.**
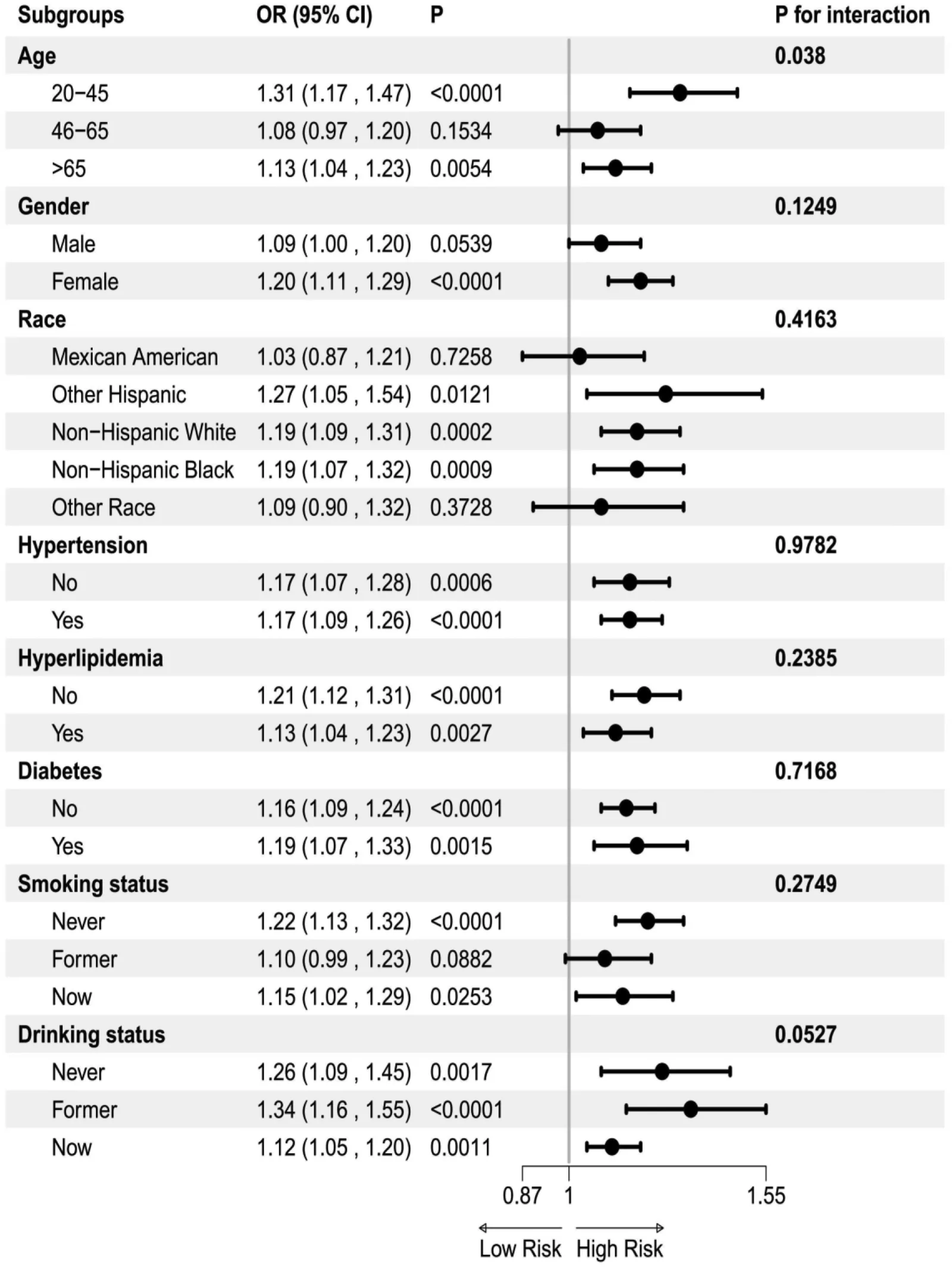
Subgroup analyses and interaction tests for the association between LN-AISI and OAB. LN-AISI = logarithmic Aggregate Index of Systemic Inflammation, OAB = overactive bladder.

Using model 3, interaction tests and subgroup analyses shown in Figure 3 systematically reveal that 8 potential modifiers – age, gender, race, smoking, drinking, hypertension, hyperlipidemia, and diabetes – differentially influence the pathological association between LN-AISI and OAB. A significant interaction effect was observed in the age subgroup, indicating that age may act as an effect modifier in the LN-AISI–OAB association. In contrast, interaction tests in other subgroups, such as diabetes and hypertension, did not reach statistical significance (*P* > .05).

### 3.5. Mediation analysis

**Figure 4.**
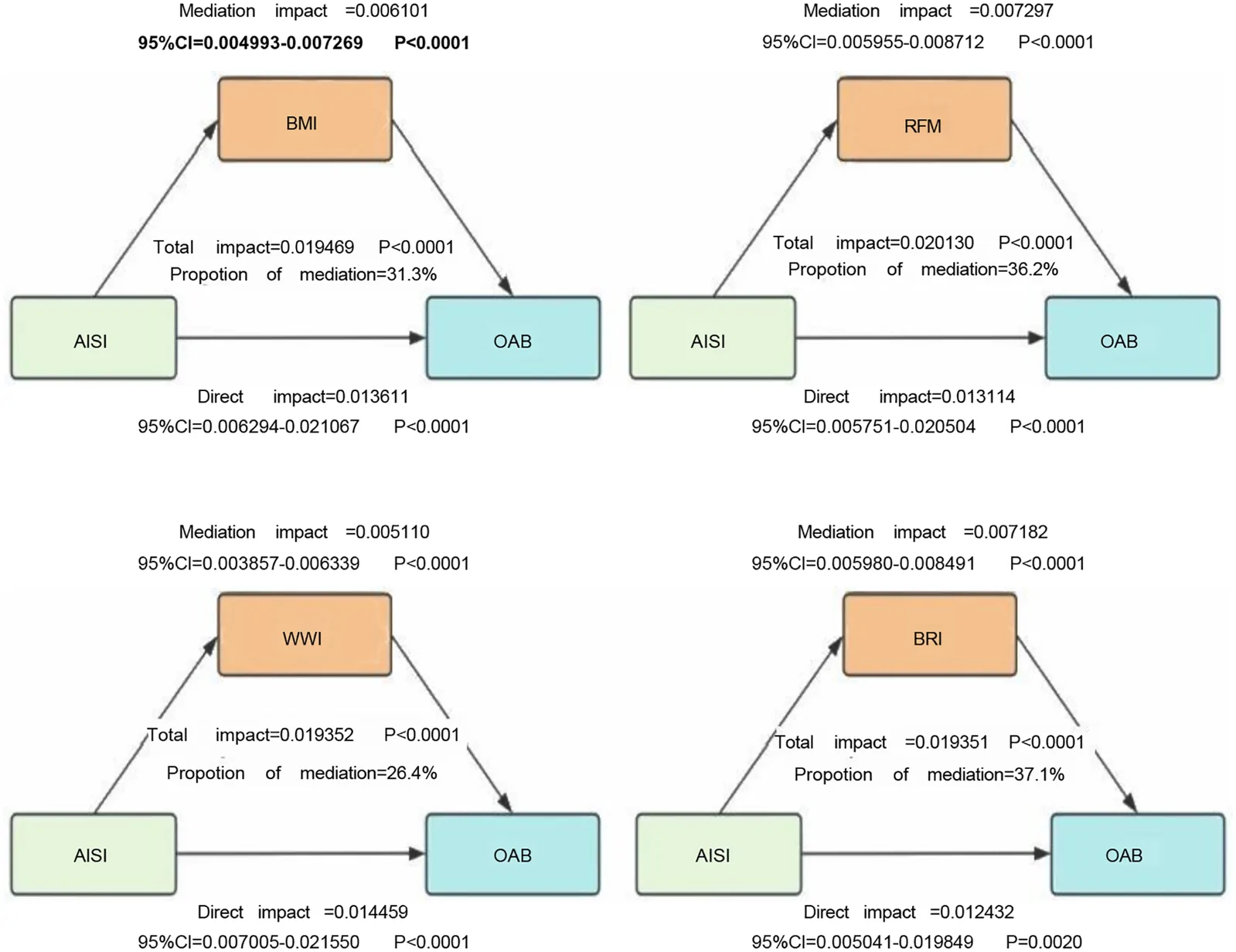
Mediation effects of body composition indices in the association between LN-AISI and OAB. LN-AISI = logarithmic Aggregate Index of Systemic Inflammation, OAB = overactive bladder.

Figure 4 illustrates the statistical mediating effects of various body composition indices in the association between LN-AISI and OAB. First, we confirmed a significant positive correlation (*P* < .05) between LN-AISI levels and 4 body measures, including BMI and RFM (see Table S3, Supplemental Digital Content 3). Second, we further investigated the associations between BMI, RFM, WWI, and BRI with OAB and found the same significant correlations (*P* < .05; see Table S4, Supplemental Digital Content 4). Finally, the results of mediation model quantification indicated that the mediation contributions of BMI, RFM, WWI, and BRI were 31.3%, 36.2%, 26.4%, and 37.1%, respectively, which suggests that adiposity indices may statistically mediate the association between systemic inflammation and OAB, highlighting a potential pathway that warrants further longitudinal and experimental validation.

## 4. Discussion

This study reveals for the first time a nonlinear association between LN-AISI and OAB based on NHANES 2009–2018 cycle data. Threshold effect analysis identified an empirical cut-point at 5.71. To the left of this threshold, variations in inflammation levels were not statistically associated with OAB odds. In contrast, on the right side of the cut-point, each unit increment of LN-AISI corresponded to a 50% increase in the odds of OAB. Subgroup analysis revealed a significant interaction for age (*P* < .05), with the association between LN-AISI and OAB being more pronounced in both younger and older age groups. No significant heterogeneity was observed across other subgroups, such as gender. Mediation analysis suggested that body composition indices such as BMI and RFM may play a statistical mediating role in the association between LN-AISI and OAB. Among these, BRI showed the highest mediation effect (37.1%), followed by RFM (36.2%), BMI (31.3%), and WWI (26.4%). These findings highlight the potential linking role of adipose metabolic pathways in the epidemiological association between LN-AISI and OAB.

The present study aligns with existing hypotheses regarding inflammatory involvement in OAB, providing population-level epidemiological evidence to support previous experimental observations, although the specific signaling pathways need to be systematically elucidated. A recent study demonstrated that patients with OAB exhibit a distinct peripheral blood inflammatory profile. Pro-inflammatory mediators, including TNF-α, MIP-1β, and SAA, were significantly elevated, while anti-inflammatory cytokines such as IL-4 and the angiogenic marker Tie2 showed reduced expression trends.^[26]^ Notably, IL-4 plays a critical role in bladder protection by regulating smooth muscle tone and suppressing inflammatory responses.^[27]^ Other studies have found that levels of nerve growth factor (NGF) and classical inflammatory markers such as CRP and MCP-1 are abnormally elevated in the urine of patients with OAB.^[28-30]^ Among them, dynamic changes in CRP and MCP-1 provide strong support for the inflammatory hypothesis of OAB.^[31,32]^ Subsequent studies on OAB treatment have similarly confirmed the involvement of inflammation in OAB symptomatology.^[33]^ However, chronic inflammatory stimulation induces alterations in bladder afferent nerve plasticity, as evidenced by increased dorsal root ganglion neuron volume and downregulation of sensory thresholds, which in turn may contribute to OAB pathology.^[34]^ In addition, elevated circulating NGF levels may enhance bladder sensory afferent signaling through activation of nociceptive receptors such as TRPV1, which in turn also produce symptoms of OAB.^[35]^ Compared with other comprehensive inflammatory indices, such as neutrophil-to-lymphocyte ratio (NLR) and systemic immunoinflammatory index (SII), the AISI is able to more comprehensively characterize systemic immunoinflammatory status by integrating multilineage immune cell parameters, such as neutrophils, monocytes, etc.^[7,36]^ In future clinical practice, a simple whole blood cell profile could potentially serve as an adjunctive biomarker to identify patients with elevated inflammatory levels, pending further longitudinal validation.

Obesity has been shown to have a significant association with OAB in numerous studies. A prospective study found that individuals with obesity had a significantly higher incidence of developing OAB. Furthermore, clinical symptom severity, subjective discomfort, and quality of life worsened progressively with increasing BMI.^[37]^ Additionally, specific patterns of body fat distribution significantly affect disease progression. For example, young women with a body fat percentage (BFP) > 32% had a 95% higher likelihood of OAB compared to those with a BFP < 32%.^[38]^ Similarly, a prospective cohort study of women with lower urinary tract symptoms also found a significant correlation between obesity and OAB.^[39]^ These findings suggest that maintaining a healthy body weight may be associated with a lower prevalence of OAB and improve overall health and quality of life. While obesity is strongly linked to OAB, its underlying biological connections involve complex pathophysiological processes. First, abnormal body fat distribution (especially centripetal obesity) creates a sustained pressure load on the bladder and pelvic floor region by triggering a significant increase in intra-abdominal pressure, which leads to compression and deformation of the bladder neck and urethral anatomy, and ultimately to an increased susceptibility to stress urinary incontinence.^[40]^ Second, metabolic syndrome – particularly that associated with abnormal fat distribution – can disrupt endocrine homeostasis and promote insulin resistance.^[41]^ Insulin-dependent diastolic responses are significantly impaired, resulting in OAB-related clinical symptoms.^[42]^ In addition, individuals with obesity have a higher prevalence of depression compared to those with normal weight.^[43]^ Patients with major depression often show significant alterations in their daily behavioral patterns, as evidenced by an increase in sedentary time or a decrease in physical activity, and their urinary habits may be altered as a result, leading to OAB-related clinical symptoms.^[44]^ As indicators of obesity, it has been shown that WWI, BMI, BRI, and RFM are associated with OAB.^[21,22,45]^ In addition, several inflammatory cytokines such as TNF, IL-1, IL-2, and IL-6 can contribute to obesity by inhibiting lipoprotein lipase biosynthesis in adipose/muscle tissues while activating the very low-density lipoprotein (VLDL) secretory pathway, which significantly elevates triglyceride concentrations in the circulatory system.^[46]^ Based on these findings, we systematically evaluated the statistical mediating role of anthropometric indicators in the association between LN-AISI and OAB using mediation modeling. The results suggested that obesity-related parameters may statistically mediate the inflammation–OAB association, providing a theoretical foundation for future investigations into preventive strategies targeting inflammation control and weight management.

In contrast to previous studies, this study focused on exploring the association between LN-AISI and OAB, and, for the first time, systematically quantified the statistical mediating role of routinely measured body parameters in this relationship. The multifactorial correlation analysis demonstrates that the clinical manifestation of OAB may be associated with an imbalance between inflammatory responses and metabolic homeostasis. Specifically, the study reveals that OAB prevalence involves complex interactions between the inflammatory regulatory network and lipid metabolic balance. This comprehensive analytical approach expands upon previous epidemiological studies. In clinical practice, physicians might eventually use body measurements and inflammatory indices to better assess OAB susceptibility, though these findings first require prospective validation.

However, several methodological limitations remain in this study. First, due to the cross-sectional observational design, it is impossible to establish temporal causality between LN-AISI and OAB. Consequently, the mediation analysis performed herein represents statistical associations rather than definitive causal pathways, and the current findings require validation through prospective cohort studies. Second, although multivariable models were used to adjust for known confounders, the influence of unmeasured latent variables cannot be ruled out. Specifically, critical variables such as menopausal status, severity of depression, and the use of specific urological or anticholinergic medications were not fully captured in the dataset, which may introduce residual confounding. Additionally, participants with missing data on the exposure, outcome, or key covariates were directly excluded, resulting in a complete-case analytical sample. Although this approach is widely used in NHANES-based research, it may introduce selection bias if the missingness was not completely at random, and the excluded participants could potentially differ systematically from those retained. Future studies employing multiple imputation or sensitivity analyses for missing data would help to address this concern. Regarding diagnostic validity, although using self-reported data from the standardized NHANES questionnaire allows for convenient large-scale assessment, subjective variation in participants’ perceptions may introduce information bias. The lack of objective urodynamic examinations to confirm the OAB diagnosis is a notable limitation. Future research should incorporate objective diagnostic tools to enhance diagnostic precision. Additionally, because the study population was drawn from the NHANES database, the generalizability of the findings may be limited in other populations, necessitating validation through multicenter and cross-national studies. Finally, although this study modeled the statistical mediating effects of anthropometric indicators, the underlying molecular mechanisms – particularly the interplay between inflammatory signaling and bladder function regulation – remain unclear, and further experimental research is needed to elucidate how body composition metrics may mediate this interaction at a mechanistic level.

## 5. Conclusions

This study revealed a nonlinear association between LN-AISI and the prevalence of OAB, with an empirical cut-point identified at 5.71. When LN-AISI exceeded this threshold, each 1-unit increase was associated with 50% higher odds of OAB (OR: 1.50, 95% CI: 1.32–1.70, *P* < .0001). Additionally, age was found to significantly modify this association. The statistical mediation model showed that anthropometric indicators had measurable statistical mediation proportions, with BMI (31.3%), RFM (36.2%), WWI (26.4%), and BRI (37.1%) mediating some of the associations, suggesting that adiposity may be statistically associated with both systemic inflammation and OAB in this population. This provides novel epidemiological evidence for understanding the complex interplay among inflammation, obesity, and OAB, and may inform future longitudinal investigations into its prevention and management.

## Author contributions

**Conceptualization:** Hao Du, Honglan Zhou.

**Data curation:** Lei Wang.

**Formal analysis:** Hao Du.

**Investigation:** Hao Du, Honglan Zhou.

**Methodology:** Hao Du, Honglan Zhou.

**Project administration:** Lei Wang.

**Resources:** Hao Du, Lei Wang, Honglan Zhou.

**Software:** Lei Wang.

**Supervision:** Lei Wang, Honglan Zhou.

**Validation:** Hao Du.

**Visualization:** Hao Du, Lei Wang.

**Writing** – **original draft:** Hao Du, Lei Wang, Honglan Zhou.

**Writing** – **review & editing:** Hao Du, Lei Wang, Honglan Zhou.








