# Supplementary material for: Association between systemic inflammation and overactive bladder: The potential mediating role of obesity-related anthropometric indices
Source: Medicine (Baltimore). 2026 Sep 11;105(37):e50574. doi: 10.1097/MD.0000000000050574 (PMC13574474; doi:10.1097/MD.0000000000050574)
Supplement: Supplementary file 1 [file medi-105-e50574-s001.docx]

Supplementary Table S1 Standards for converting NHANES symptom frequencies to OABSS scores

| **Urge urinary incontinence frequency**  **(from NHANES)** | **Urge urinary incontinence score**  **(according to OABSS)** | **Nocturia frequency (from NHANES)** | **Nocturia score (according to OABSS)** |
| --- | --- | --- | --- |
| Never | 0 | 0 | 0 |
| Less than once a month | 1 | 1 | 1 |
| A few times a month | 1 | 2 | 2 |
| A few times a week | 2 | 3 | 3 |
| Every day and/or night | 3 | 4 | 3 |

NHANES：National Health and Nutrition Examination Survey

OABSS：Overactive Bladder Symptom Score
