# Supplementary material for: Association between systemic inflammation and overactive bladder: The potential mediating role of obesity-related anthropometric indices
Source: Medicine (Baltimore). 2026 Sep 11;105(37):e50574. doi: 10.1097/MD.0000000000050574 (PMC13574474; doi:10.1097/MD.0000000000050574)
Supplement: Supplementary file 2 [file medi-105-e50574-s002.docx]

Supplementary Table S2 Description of covariates

| Covariates | Description in NHANES |
| --- | --- |
| Gender | Male and Female |
| Race | Mexican American, other Hispanic, non-Hispanic white, non-Hispanic black, other race |
| Educational level | less than high school, high school, more than high school |
| Marital status | never married, widowed/divorced/separated, married/living with a partner |
| Smoking | Smoking status was grouped into never smoker (defined as <100 cigarettes in a lifetime) ,current smoker (defined as ≥100 cigarettes in a lifetime), and former smoker (defined as ≥100 cigarettes and had quit smoking) |
| Drinking status | Drinking status was grouped into never drinker(defined as<12 alcohol drinks in a lifetime),current drinker(defined as≥12 alcohol drinks in a lifetime),and former drinker(defined as≥12 alcohol drinks in a lifetime and had quit drinking) |
| Diabetes | Diabetes was defined as a history of previous diabetes, now taking insulin,now taking diabetic pills to lower blood sugar,HbA1c level ≥6.5%, or fasting blood glucose level ≥126 mg/dL |
| Hypertension | The diagnostic criteria consist of self-reported hypertension history, the utilization of antihypertensive medication, a systolic blood pressure (SBP) ≥ 140mmHg, or a diastolic blood pressure (DBP) ≥ 90mmHg |
| Hyperlipidemia | Diagnostic criteria were that the participant was told by a physician or other health professional that the participant had a high blood cholesterol level, was currently using lipid-lowering medication to reduce blood lipids, and had a total cholesterol level of ≥240 mg/dL |
