# Supplementary material for: Association between systemic inflammation and overactive bladder: The potential mediating role of obesity-related anthropometric indices
Source: Medicine (Baltimore). 2026 Sep 11;105(37):e50574. doi: 10.1097/MD.0000000000050574 (PMC13574474; doi:10.1097/MD.0000000000050574)
Supplement: Supplementary file 3 [file medi-105-e50574-s003.docx]

Supplementary Table S3 Associations between the LN-AISI and body measurements

|  | OR,(95%CI),P value |
| --- | --- |
| Outcome:BRI |  |
| LN-AISI | 0.57 (0.50, 0.64) <0.0001 |
| Outcome:WWI |  |
| LN-AISI | 0.18 (0.15, 0.20) <0.0001 |
| Outcome:RFM |  |
| LN-AISI | 1.34 (1.18, 1.50) <0.0001 |
| Outcome:BMI |  |
| LN-AISI | 1.48 (1.28, 1.69) <0.0001 |

BRI:body roundness index;WWI:weight-adjusted waist index;RFM:relative fat mass;BMI:body mass index;LN-AISI:Natural logarithmically transformed the aggregate index of systemic inflammation
