# Supplementary material for: Association between systemic inflammation and overactive bladder: The potential mediating role of obesity-related anthropometric indices
Source: Medicine (Baltimore). 2026 Sep 11;105(37):e50574. doi: 10.1097/MD.0000000000050574 (PMC13574474; doi:10.1097/MD.0000000000050574)
Supplement: Supplementary file 4 [file medi-105-e50574-s004.docx]

Supplementary Table S4 Associations between body measurements and OAB

| Outcome：OAB | OR,(95%CI),P value |
| --- | --- |
| BRI | 1.23 (1.21, 1.25) <0.0001 |
| WWI | 2.17 (2.04, 2.31) <0.0001 |
| RFM | 1.07 (1.06, 1.08) <0.0001 |
| BMI | 1.05 (1.05, 1.06) <0.0001 |

BRI:body roundness index;WWI:weight-adjusted waist index;RFM:relative fat mass;BMI:body mass index;OAB:overactive bladder
